# Polyphasic Approach Including MALDI-TOF MS/MS Analysis for Identification and Characterisation of *Fusarium verticillioides* in Brazilian Corn Kernels

**DOI:** 10.3390/toxins8030054

**Published:** 2016-02-24

**Authors:** Susane Chang, Mariele Porto Carneiro-Leão, Benny Ferreira de Oliveira, Cristina Souza-Motta, Nelson Lima, Cledir Santos, Neiva Tinti de Oliveira

**Affiliations:** 1 Postgraduate Program in Fungal Biology, Department of Mycology, Federal University of Pernambuco, Av. Prof. Nelson Chaves, s/n, Cidade Universitária, Recife 50670-420, PE, Brazil; susicchang@hotmail.com (S.C.); mariele_carneiro@hotmail.com (M.P.C.-L.); benny.oliveira74@hotmail.com (B.F.d.O.); cristina.motta@ufpe.br (C.S.-M.); netinti@hotmail.com (N.T.d.O.); 2 CEB-Centre of Biological Engineering, Micoteca da Universidade do Minho, University of Minho, Campus of Gualtar, 4710-057 Braga, Portugal; nelson@ie.uminho.pt; 3 Department of Chemical Sciences and Natural Resources, CIBAMA, BIOREN-UFRO, Faculty of Engineering and Sciences, Universidad de La Frontera, Av. Francisco Salazar, 01145 Temuco, Chile

**Keywords:** mycotoxin, fumonisins, mycotoxigenic fungi, genetic study

## Abstract

*Fusarium verticillioides* is considered one of the most important global sources of fumonisins contamination in food and feed. Corn is one of the main commodities produced in the Northeastern Region of Brazil. The present study investigated potential mycotoxigenic fungal strains belonging to the *F. verticillioides* species isolated from corn kernels in 3 different Regions of the Brazilian State of Pernambuco. A polyphasic approach including classical taxonomy, molecular biology, MALDI-TOF MS and MALDI-TOF MS/MS for the identification and characterisation of the *F. verticillioides* strains was used. Sixty *F. verticillioides* strains were isolated and successfully identified by classical morphology, proteomic profiles of MALDI-TOF MS, and by molecular biology using the species-specific primers VERT-1 and VERT-2. *FUM1* gene was further detected for all the 60 *F. verticillioides* by using the primers VERTF-1 and VERTF-2 and through the amplification profiles of the ISSR regions using the primers (GTG)_5_ and (GACA)_4_. Results obtained from molecular analysis shown a low genetic variability among these isolates from the different geographical regions. All of the 60 *F. verticillioides* isolates assessed by MALDI-TOF MS/MS presented ion peaks with the molecular mass of the fumonisin B1 (721.83 g/mol) and B2 (705.83 g/mol).

## 1. Introduction

Corn (*Zea mays* L.) is the most intensively cultivated cereal crop in the world, mainly due to its adaptive nature. It can be cultivated in both tropical and temperate climates and from sea level up to *ca.* 3.5 km. The economic importance of corn is also reflected in its diverse use. While most harvested corn is used to prepare animal feed, use of corn derivatives is quite important in food production. In Brazil, corn is an important food source for many people living in the semi-arid area of the Northeastern Region [1].

Fungi are ubiquitous microorganisms known to produce a wide variety of secondary metabolites, which play important role in diversification and adaptation of these microorganisms to various ecological niches, including plants cultivated for food and feed production, such as the corn plant and kernels [2,3,4,5,6,7,8]. Secondary metabolites have attracted huge interest of various researchers due to their potential for biotechnological applications such as development of drugs, cosmetics, food, and others [9]. However, one of the main concerning related with some fungal secondary metabolites is their toxicity.

*Fusarium verticillioides* Sacc. Niremberg (= *Fusarium moniliforme* Sheldon) is a non-obligatory parasite that commonly occurs in corn plants. It can cause damage to the roots and stem and spoilage in the produced corn kernels. Infection by this fungus is often asymptomatic. This species produces a set of mycotoxins and is considered one of the most globally significant sources of contamination by fumonisins in food products, especially in corn kernels.

There are 28 known chemical compounds of natural occurrence produced by filamentous fungi that are analogues of fumonisins. These compounds can be divided into four groups, identified as the A, B, C and P series. From a toxicological point of view, the B series (FB_1_, FB_2_ and FB_3_) is the most relevant one, and fumonisins belonging to this series are common natural contaminants in corn [10].

Genetic studies suggest that three *loci* in *F. verticillioides* are closely related to fumonisin synthesis. In this case, *FUM*1 gene would be responsible for controlling the productive capacity of fumonisins by the fungus, while *FUM*2 and *FUM*3 would be responsible for controlling the hydroxylation of the C-10 and C-05 in the fumonisin molecule. Although this taxon is the most prolific producer of fumonisins, some *F. verticillioides* isolates do not present the *FUM*1 gene. This means that *F. verticillioides* strains missing the *FUM*1 gene are not potential producers of fumonisins [10].

The *FUM*1 gene, and other genes of this biosynthetic pathway, has been detected in *F. verticillioides* isolates through the use of PCR gene amplification techniques [11,12,13,14]. In order to detect the presence of fungi in plant materials, specific primers in PCR reactions have widely been used. This technique is rapid, relatively easy to perform, quite sensitive, and can be used at wide or limited scales to examine the genetic structures of fungal populations [12,15].

A number of molecular techniques have been employed to characterise different species of *Fusarium*. It includes PCR based on species-specific primers and the inter-simple sequence repeat (ISSR), which are molecular markers to detect genetic variability among populations [12,15,16,17].

In addition, matrix-assisted laser desorption/ionisation time-of-flight (MALDI-TOF) is a mass spectrometry technique that has demonstrated high potentiality on the identification of filamentous fungi at species and, in some specific cases, at strain level [18]. One of the most interesting advantages of the technique is the possibility of analysing the intact fungal cell generating specific molecular mass profiles [5,19,20,21,22].

As previously described in different studies, MALDI-TOF MS analysis for fungal identification is based on specific molecular masses that are mainly constituted by ribosomal proteins [5,20,22]. The mass peaks generated by the analysis of these ribosomal proteins are observed in a mass range between 2 and 20 kDa. Also, compounds such as polysaccharides, lipids, phospholipids, and chitin, among others, can similarly be found in the fungal cell. All of these are very useful in fungal identifications by mass spectrometry [5,18,19,21,23,24].

Studies have demonstrated the high potentiality of the technique for species and strain identification within the *Fusarium* genus [24,25,26]. Recently, Santos *et al.* [27] used MALDI-TOF MS to establish a multistep identification of *Fusarium guttiforme* and its antagonist *Trichoderma asperellum* infecting pineapple side shoots. In addition, Rodrigues *et al.* [18] evaluated the MALDI-TOF MS technique regarding its capability for the species identification of fungal strains belonging to the genus *Aspergillus* section *Flavi* and their characterisation as aflatoxigenic or non-aflatoxigenic strains. According to the authors, the method was useful for fungal species differentiation. However, it was not able to differentiate strains according their mycotoxigenic potential.

The presence of fungal species such as *F. verticillioides*, which is capable of producing mycotoxins in agricultural commodities, is a constant threat to both human and animal health. Efficient techniques of control that must include studies of inter- and intra-specific variations of this taxon and their capacities to produce mycotoxins are required [10].

MALDI TOF tandem mass spectrometry (MS/MS) has been used as an important analytical tool in the analysis of low molecular weight physiological or therapeutic molecules of interest, such as amino acids and other pharmaceuticals [28]. However, the application of MALDI-TOF MS/MS for the detection of fumonisins has not been addressed yet in the scientific literature.

As corn kernels are one of the main food and feed resources in the Brazilian Northeastern Region, and based on the lack of studies and information focusing the occurrence of mycotoxigenic *F. verticillioides* strains in this food and feed commodity produced in the Brazilian State of Pernambuco, the aim of the present study was to investigate the presence of mycotoxigenic *F. verticillioides* strains isolated from corn kernels in 3 different Regions of this State. For this propose, a polyphasic approach including classical taxonomy, molecular biology, MALDI-TOF MS and MALDI-TOF MS/MS for the identification and characterisation of the *F. verticillioides* strains was used.

## 2. Results

### 2.1. Isolation and Identification of Fungal Strains

Sixty *F. verticillioides* isolates were obtained from the corn kernels evaluated in the present study (Table 1). No difference in the fungal growth and isolation was observed between PDA and DG18 media. Each isolate presented typical macro- and microscopic characteristics observed for the *F. verticillioides* species (data not shown), as previously described elsewhere [29].

**Figure 1:**
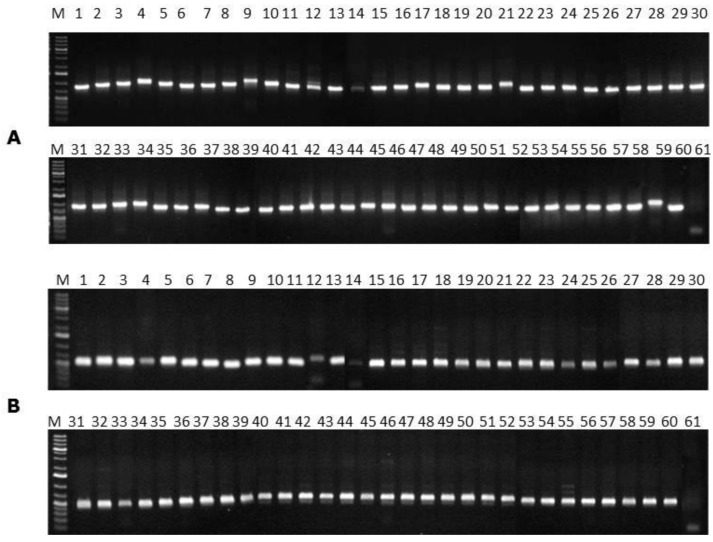
Molecular biology data for (**A**) fungal species identification by amplification profiles using the primers VERT-1 and VERT-2 and (**B**) *FUM*1 gene detection using the primers VERTF-1 and VERTF-2. Columns (M) 1 Kb Plus DNA Ladder; (1 to 60) *F*. *verticillioides* isolates; (61) *Penicillium glabrum* URM 3585.

In order to confirm the species identification, all 60 morphologically identified *F*. *verticillioides* isolates were subjected to PCR reactions. The primers VERT-1 and VERT-2 were used. All of the isolates presented 800 bp amplification products as depicted in the Figure 1A. These amplification products were not seen in the negative control Penicillium glabrum URM 3585 (Figure 1A).

According to the data obtained by the statistical analysis of relatedness based on proteomic profiles by MALDI-TOF MS (data not shown), all isolates were correctly grouped and identified at the species level, confirming their identification generated by classical taxonomy and molecular biology approaches. In addition, no grouping concerning the geographical origin was observed among the evaluated strains through the MALDI-TOF MS results.

### 2.2. Detection of FUM1 Gene

The *F*. *verticillioides* isolates were subjected to PCR analyses using the primers VERTF-1 and VERTF-2. Fragments of *ca.* 500 bp were obtained, which confirmed the presence *FUM1* gene. In addition, no amplification products were detected for the *Penicillium glabrum* URM 3585 (Figure 1B).

**Figure 2:**
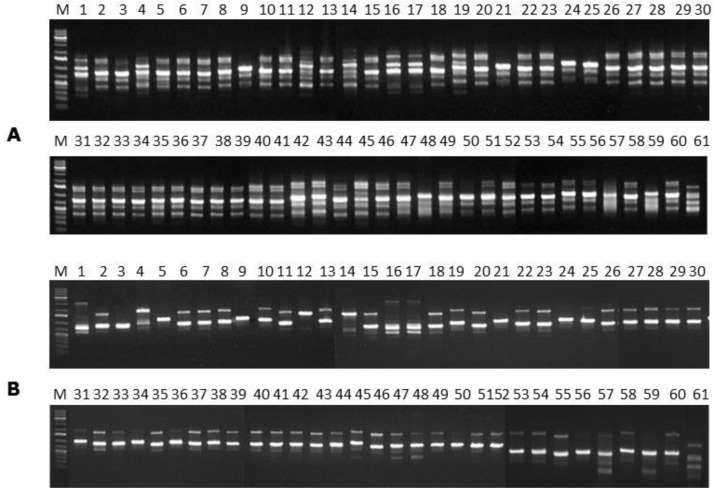
ISSR amplification profiles obtained using the primers (**A**) (GTG)_5_ and (**B**) (GACA)_4_. Columns: (M) 1 Kb Plus DNA Ladder; (1 to 60) *F. verticillioides* isolates; (61) *Penicillium glabrum* URM 3585.

**Figure 3:**
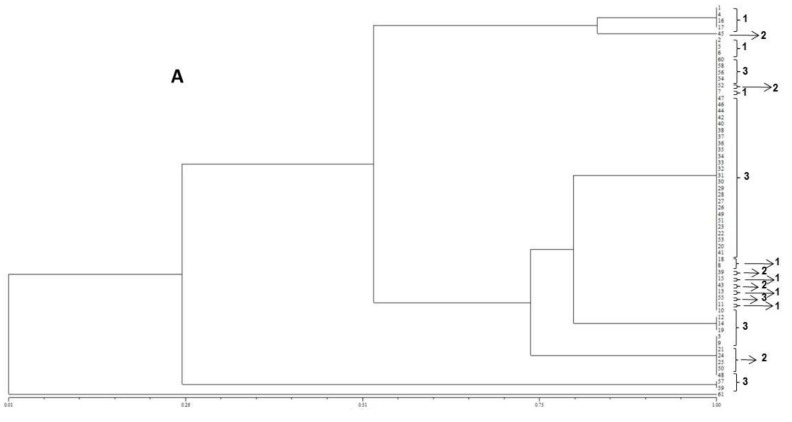

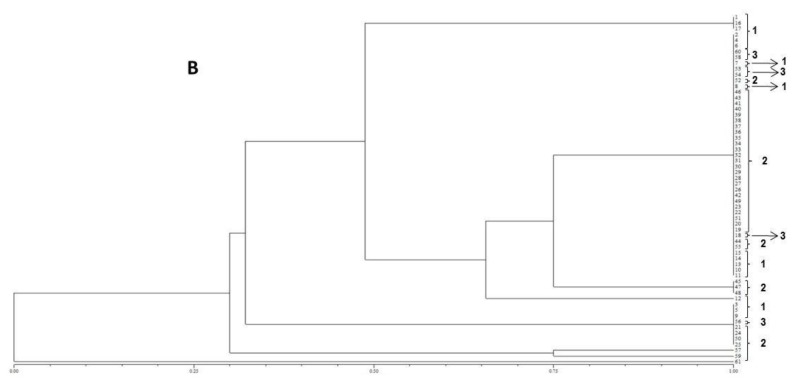
Dendrogram constructed using UPGMA, utilizing the Jaccard coefficient (J) based on the amplification profiles of the ISSR region using the primers (**A**) (GTG)_5_ and (**B**) primer (GACA)_4_; Strains from 1: Zona da Mata; 2: Sertão; and 3: São Francisco.

For each *F. verticillioides* isolate evaluated the amplification profiles of the ISSR regions using the primer (GTG)_5_ generated from 1 to 6 fragments with molecular weights varying from approximately 300 to 1500 bp (Figure 2A). After statistical analysis, 5 distinct groups with 100% similarity in terms of their fragment sizes were obtained (Figure 3A). The isolate 45 was the only one statistically outgrouped from the others, as it can be observed in Figure 3A.

The ISSR amplification profiles based on the primer (GACA)_4_ for the 60 *F. verticillioides* strains evaluated generated from 1 to 4 fragments for each fungal strain, with molecular weights varying from approximately 300 to 2000 bp (Figure 2B). The markers formed 4 distinct groups with 100% similarity, separating three isolates only, namely 12, 57 and 59 (Figure 3B).

### 2.3. Detection of Fumonisins by MALDI-TOF MS/MS

**Figure 4:**
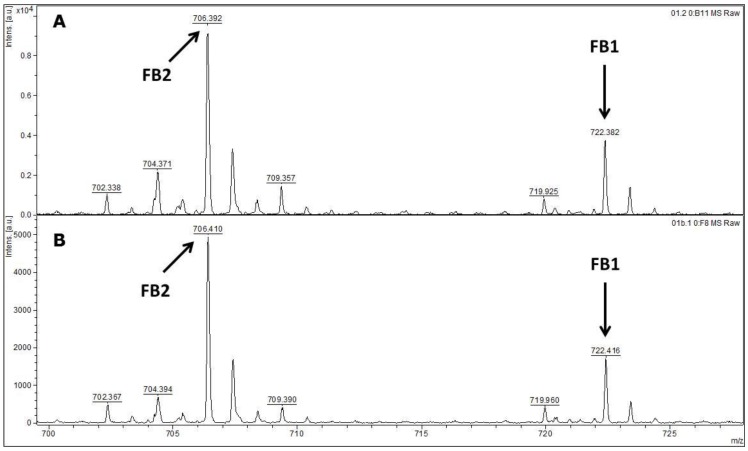

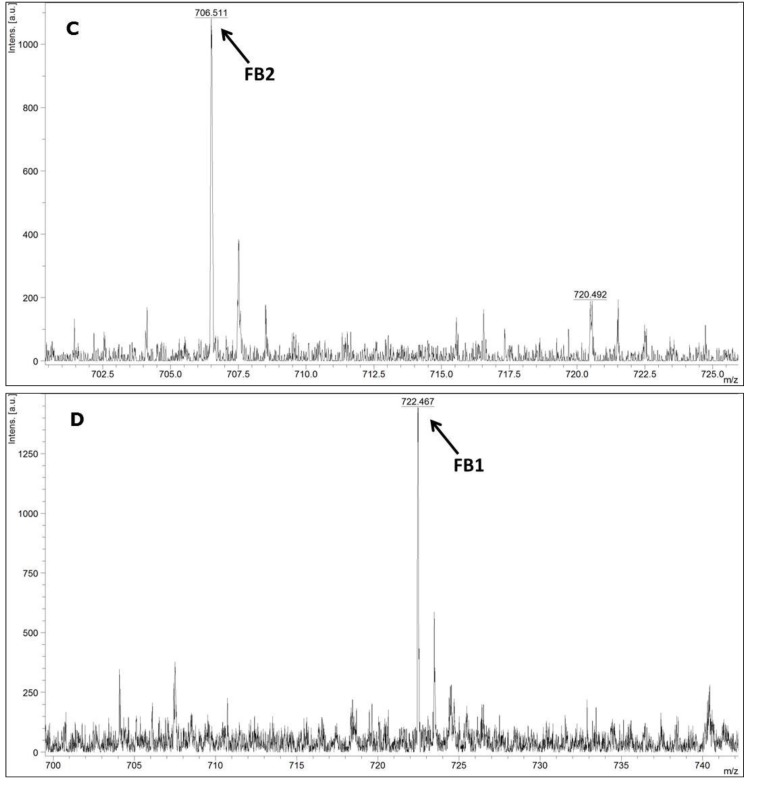
MALDI-TOF MS/MS spectra with secreted fumonisins B1 (721.83 g/mol) and B2 (705.83 g/mol) on corn-based medium culture for the isolates 1 (**A**) and 3 (**B**) of *F. verticillioides*; (**C**) and (**D**) are the standards for fumonisin B1 and B2, respectively.

All of the 60 *F. verticillioides* isolates assessed by MALDI-TOF MS/MS presented ion peaks with the molecular mass of the fumonisin B1 (721.83 g/mol) and B2 (705.83 g/mol). Figure 4 shows the results for both mycotoxins detections for the isolates 1 and 3. Moreover, in addition to the fumonisin B1 and B2, the other *F. verticillioides* isolates presented a variable number of peaks in their mass spectra according to their capability to produce other kind of metabolites (data not shown).

## 3. Discussion

The results obtained herein demonstrated the appropriateness of the species-specific primers VERT-1 and VERT-2 used for confirming the identification of *F. verticillioides* isolates previously identified by classical morphology. All of the evaluated isolates presented 800 bp amplification products, as expected for this species. In fact, phenotypic methods are still relevant in daily routing analysis in a laboratory of mycology, but these methods are frequently associated with misidentification of some taxa. This is specially certainty for species belonging to the *Fusarium* genus, as their micromorphology can vary according to the culture medium employed.

Due to morphological variations within the genus *Fusarium* and due to the fact that some species of the genera *Cylindrocarpon* and *Acremonium* are also able to produce fusiform and multicellular conidia, similar to those produced by *Fusarium* species, molecular methods can significantly support confirmation of the taxonomy of that genus [27,30].

The data obtained in the present study are in agreement with the data presented by Dissanayake *et al.* [11], who evaluated fungal strains isolated from seeds and plants of Welsh Onion (*Allium fistulosum*) in Japan. Authors successfully used the species-specific primers VERT-1 and VERT-2 to differentiate strains of *F*. *verticillioides* and further used the primers PRO1 (5′-CTTTCCGCCAAGTTTCTTC-3′) and PRO2 (5′-TGTCAGTAACTCGACGTTGTTG-3′) to differentiate strains of *F*. *proliferatum*.

A number of molecular biology techniques have been used to characterise different species of *Fusarium*, including species-specific PCR primers. In order to prevent the early food contamination by fumonisins, González-Jáen *et al.* [17] evaluated the species-specific primers VERT-1 and VERT-2 as fast and reliable system for the detection of *F*. *verticillioides* able to produce these fungal secondary metabolites.

In addition, in order to identify *Fusarium* species from corn kernels in India, Sreenivasa *et al.* [15] used the primer VERT-R (5′-CGACTCACGGCCAGGAAACC- 3′) combined with the primer VERTF-1 (5′-GCGGGAATTCAAAAGTGGCC-3′). The authors reported that 83 strains out of the 103 evaluated were identified at the species level as *F. verticillioides*.

Some *F. verticillioides* strains have been described as non-fumonisin producers [12,14]. Sánchez-Rangel *et al.* [13] evaluated 34 *F. verticillioides* strains isolated from corn in Mexico and found a correlation between the fumonisins production and the presence of the *FUM1* gene. The authors conclude that almost all of the *F. verticillioides* strains that were fumonisin producers presented the *FUM1* gene.

In fact, the *FUM1* gene has widely been used as a molecular marker for the determination of mycotoxigenic *F. verticillioides* strains. Maheshwar *et al.* [12] used successfully the primers VERT-1 and VERT-2 to distinguish fumonisin producers from non-producers strains of *F. verticillioides* in 90 samples of stored paddy (*Oryza sativa* L.), collected from different geographical regions of Karnataka, India.

In addition, Silva *et al.* [14] analysed 27 *F. verticillioides* strains isolated from corn and sorghum in Brazil. According to the authors, among the evaluated strains, 6 presented the *FUM1* gene. Karthikeyan *et al.* [31] evaluated 82 strains belonging to different *Fusarium* species isolated from rice samples contaminated with fumonisins. In order to distinguish fumonisin producers from non-producers strains authors assessed fungal isolates by using PCR analyses through the primers VERTF-1 and VERTF-2. Twenty-one out 46 *F. verticillioides* strains presented amplification for the *FUM1* gene.

In the present study, the *FUM1* gene was detected for all of the *F. verticillioides* strains assessed, confirming their potentials to produce fumonisins. The statistical analysis of the gels assembled using the primers (GTG)_5_ and (GACA)_4_ point out to a high genetic homogeneity among the different isolates. In terms of the geographical origins no groupings were observed for the assessed isolates. However, high levels of similarity among isolates were observed when ISSR primers were used. Furthermore, the (GTG)_5_ primer showed the highest genetic variability among them.

Few works have reported the use of ISSR primers for evaluation of *F. verticillioides* strains. Lima [32] reported that the (GTG)_5_ primer was efficient for detecting differences among *Metarhizium anisopliae* strains. Based on data obtained by the (GACA)_4_ primer, the author found significant genetic variability among mutants and wild types strains belonging to the genus *Metarhizium*. According to the author, the genetic variability found among the assessed strains allows their adaptation to the different hosts and wide geographic regions. In addition, Bayraktar *et al.* [16] evaluated 74 strains of *F. oxysporum* f. sp. *ciceris* using 20 ISSR primers. The authors reported that the genetic variability of the evaluated strains was greater according to the geographical regions of each evaluated strain.

Several studies have been proven the capability of MALDI-TOF MS to identify fungal species belonging to different fungal genus. MALDI-TOF MS has been proven to be useful for the identification of filamentous fungi at either the species or strain level [18,19,33]. Moreover, previous studies have demonstrated the usefulness of MALDI-TOF MS-based fingerprinting methodology for the identification of species of *Fusarium* [24,25,26,27].

By comparison with molecular biology data, MALDI-TOF MS has herein been shown to be sensitive (100% correct identification) for *F. verticillioides* identification at the species level. The MALDI-TOF MS technique can thus be regarded as an additional step to the polyphasic scheme of identification of this fungal species. Furthermore, it is an objective and fast analytical methodology, and inexpensive in terms of labour and consumables when compared to other molecular biological techniques. As a consequence, it is suitable for applications which have particular high-throughput needs [18]. According to the results obtained in the present study, no differences according to the geographical origin of the fungal strains were observed, which is in accordance with the molecular biology data obtained.

MALDI-TOF MS/MS was used in the present study to evaluate the production of fumonisin B1 and B2. These mycotoxins have been usually evaluated by other analytical techniques, such as HPLC or liquid chromatography coupled with mass spectrometry (e.g., LC-MS/MS) [34,35]. According to the data available in the scientific literature, there is no information about the use of MALDI-TOF MS/MS for the detection of fumonisin B1 and B2 in moistened ground corn samples obtained from corn kernels, which make this study the first report on the use of this analytical technique for this purpose. The results obtained herein for mycotoxin detection by MALDI-TOF MS/MS are in agreement with the data obtained by molecular biology based on the *FUM1* gene amplification, where all of the 60 strains evaluated were characterised as potential fumonisin B1 and B2 producers.

In conclusion, the results obtained in the present work point to a low genetic variability among the *F. verticillioides* strains isolated from corn kernels in different places in the 3 assessed Regions of the Brazilian State of Pernambuco. Furthermore, it was not possible to distinguish the *F. verticillioides* strains based on their origins, which suggest a homogeneous and very well adapted population. The MALDI-TOF technique was as good as molecular biology for the identification of *F. verticillioides*. In addition, MALDI-TOF MS/MS was reliable for the detection of fumonisin B1 and B2. Moreover, the polyphasic approach based on the different employed methodologies leads to a reliable final species identification. It is an important approach when food safety is the main subject, as is the case in the production of corn kernels.

## 4. Experimental Section

### 4.1. Corn Kernel Samples

**Figure 5:**
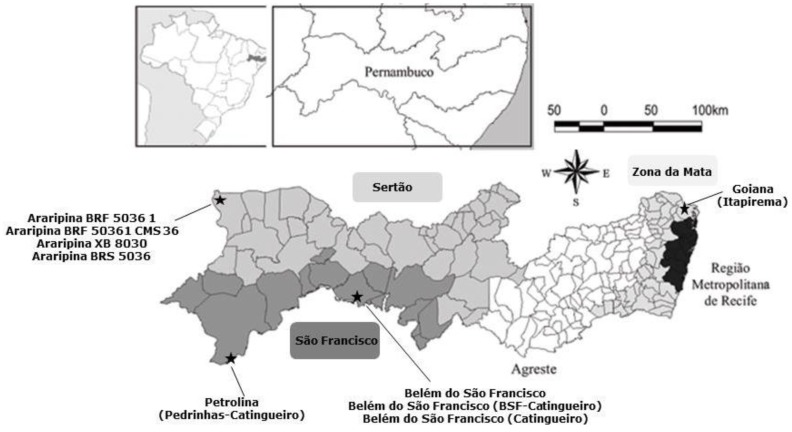
Map of Pernambuco State with information of the 3 Regions and harvest locations for the corn cobs evaluated in the present study (Adapted from [36]).

A total of 40 corn cobs were freshly harvested directly from the corn plant from July to August 2014 from different agriculture fields distributed among the following regions of Pernambuco State: São Francisco (15 corn cobs), Sertão (15 corn cobs) and Zona da Mata (10 corn cobs) (Figure 5). Samples were provided by the Experimental Stations of the Agronomic Institute of Pernambuco-IPA and by local farmers.

### 4.2. Fungal Isolation and Identification

For the sample selection the phytosanitary of corn cobs was taken into consideration. Only visually heathy corn cobs free of fungal colonies, insects and lesions were selected. After selection, the corn cobs were stored in plastic bags, labelled, stored at −4 °C and transferred to the Laboratory of Fungal Molecular Genetics (Department of Mycology, Federal University of Pernambuco, Recife, Brazil).

A total of 200 hundred corn kernels randomly collected from the corn cobs harvested from the 3 different regions, as follow: São Francisco (15 corn cobs, 5 corn kernels per each cob), Sertão (15 corn cobs, 5 corn kernels per each cob) and Zona da Mata (10 corn cobs, 5 corn kernels per each cob). The corn kernels were surface-sterilised in 70% ethyl alcohol for 30 s, in 1.5% sodium hypochlorite for 5 min, rinsed thrice in sterile distilled water for 30 s, and subsequently dried with sterile paper towels. Corn kernel samples were further dried at room temperature under sterile conditions for 24 h. The kernels were then transferred to Petri dishes (10 kernels per plate) containing potato-dextrose-agar medium (PDA) supplemented with chloramphenicol (100 mg·L^−1^) and Dichloran-Glycerol agar-base (DG18, CM0729, Oxoid, Acumedia, Lansing, MI, USA) supplemented with chloramphenicol (100 mg·L^−1^). Samples were incubated at room temperature (25 ± 2 °C) for 5 days.

The numbers of fungal colonies were further determined and those fungal colonies indicative of *Fusarium* sp. were sub-cultured in PDA and Dichloran-Glycerol (DG18) Agar Base plates and incubated for 5 days at 26 °C. Monospore cultures of *Fusarium* species were subsequently evaluated by their macro- and micro-morphologies. Overall, macroscopic characteristics of each isolate, such as its colony colour and colony diameter, as well as the microscopic characteristics such as the shape and sizes of both micro- and macro-conidia, conidiophores, and phialides were assessed. Final fungal species identification followed the taxonomic keys and guides available for *Fusarium* species [29].

### 4.3. Mycelium Growth and DNA Extraction

*Fusarium verticillioides* isolates were inoculated on PDA plates and incubated at 25 °C for 7 days. Genomic DNA was extracted from the mycelium of each *F. verticillioides* isolate. *Penicillium glabrum* URM 3585 was used as negative control. The method for genomic DNA extraction was based on bead-beating at an agitation velocity of 5.0 m/s for 60 s in a microtube containing *ca.* 65 mg of mycelium and 700 μL of 2% CTAB buffer, pH 8.0 (previously heated to 65 °C).

Microtubes containing powdered fungal mycelium were incubated at 65 °C for 1 h and subsequently centrifuged at 12,000 rpm for 10 min at 24 °C. Each supernatant was transferred to a sterile microtube and then 650 μL of chloroform/isoamyl alcohol (24:1) solution was added. The solution was then centrifuged at 10,000 *g* and the aqueous phase was transferred to a sterile microtube, in which an equal volume of absolute isopropanol previously cooled at −20 °C was added.

In order to precipitate the nucleic acids, the final solution was incubated at −20 °C for 60 min. Samples were centrifuged at 10,000 *g* for 10 min and supernatants were discarded. Sediments were subsequently suspended and washed in 1 mL of 70% ethanol. Mixtures were centrifuged at 10,000 *g* for 5 min and supernatants were discarded. Samples were incubated at 37 °C up to the total evaporation of alcohol. The DNA samples were suspended with 50 μL of Tris-EDTA buffer (pH 8.0). Samples were kept at 4 °C for 24 h and subsequently stored at −20 °C up to their analysis.

### 4.4. Molecular Biology

In order to identify *F. verticillioides* at species level, the primers VERT-1 (5′-GTCAGAATCCATGCCAGAACG-3′) and VERT-2 (5′-CACCCGCAAATCCATCAG-3′) were used. In addition, the detection of the *FUM1* gene was carried out by using the primers VERTF-1 (5′-GCGGGAATTCAAAAGTGGCC-3′) and VERTF-2 (5′-GAGGGCGCGAAACGGATCGG-3′), as previously described elsewhere [37].

The amplification reactions were undertaken in a final volume of 25 µL, containing: 20 mM Tris–HCl buffer (pH 8.4), 50 mM KCl, 0.75 mM MgCl_2_, 2 mM dNTP, 20 pmol of the primer, 3 U/μL of *Taq* DNA polymerase, and 25 ng of DNA [12]. The Techne TC-512 thermocycler with the following programmed cycles was used: 94 °C for 1 min for initial denaturation, followed by 35 cycles of denaturation for 1 min at 94 °C, annealing at 60 °C for 1 min, extension at 72 °C for 1 min, and a final extension at 72 °C for 5 min. The amplified products and the 1 Kb plus DNA ladder (Invitrogen Life Tecnologies, Carlsbad, CA, USA) were stained with *GelRed*^TM^ (Biotium, Hayward, CA, USA), separated by electrophoresis (Hoefer, Holliston, MA, USA) on 1.0% agarose gel at 3 V·cm^−1^ in TAE (1×) (pH 8.0) running buffer and visualised in an ultraviolet light transilluminator (Vilber Lourmat, Suarlée, Belgium). Images were recorded using a Sony digital camera (7.2 effective mega pixels) (Sony, Toquio, Japan).

### 4.5. Analyses of Genetic Variability

In order to evaluate the genetic variability of the *F*. *verticillioides* strains, PCR reactions using the ISSR primers (GTG)_5_ [38] and (GACA)_4_ [39] were used. Amplification reactions were performed in a final volume of 25 µL using the following contents: 20 mM Tris-HCl buffer (pH 8.4), 50 mM KCl, 0.75 mM MgCl_2_, 0.25 mM dNTP, 0.25 mM of the primer, 0.4 U of *Taq* DNA polymerase (Operon Technologies, Alameda, CA, USA), and 25 ng of DNA.

Amplification reactions were performed in a Techne TC-512 thermocycler (Analitica, São Paulo, Brazil) with the following programmed cycles: an initial denaturation step at 93 °C for 5 min, 40 cycles of 20 s at 93 °C, 45 s at 55 °C and 90 s at 72 °C, followed by a final extension at 72 °C for 6 min. The amplified products and the 1 Kb plus ladder DNA (Invitrogen Life Tecnologies) were stained with GelRed^TM^, separated by electrophoresis in 1% agarose gel at 4 V′cm^−1^ in the running buffer TAE (1×) (pH 8.0). The gel was visualised after electrophoresis by using an ultraviolet transilluminator, and registered using a Sony digital camera (7.2 effective mega pixels).

### 4.6. Statistical Analyses NTSYS-PC

The data obtained from the PCR amplifications using (GTG)_5_ and (GACA)_4_ primers were analysed using the Numerical Taxonomy System of Multivariaty Programs—NTSYS-PC [40]. The data were introduced in the form of binary variables, with the number 1 meaning the presence of a band, and the number 0 (zero) its absence. A matrix of similarity was then constructed by using Jaccard coefficient (J) [41]. The matrix of similarity was used to construct a dendrogram based on the Unweighted Pair Group Method with Arithmetical Average (UPGMA).

### 4.7. Fungal Identification by MALDI-TOF MS

Seven-day-old spores of each *Fusarium* suspended in 0.5 mL of 0.2% agar were used for inoculation on a single-point 6 cm PDA plate. All isolates were incubated for 3 days at 25 °C. Approximately 1 μg of mycelium or mycelium/spores mixture from the growing edge colony was transferred directly from the culture plate to the 48-well MALDI flex target plate (Flexi-Mass™, Shimadzu Biotech, Manchester, UK). Immediately, 0.5 μL matrix solution (75 mg/mL 2,5-dihydroxybenzoic acid [DHB] in ethanol/water/acetonitrile (1:1:1) with 0.03% trifluoroacetic acid [TFA]) was added and mixed gently. *Escherichia coli* DH5α was obtained from the Public Portuguese Culture Collection *Micoteca da Universidade do Minho-MUM* and its cells were used for *in situ* extraction of ribosomal proteins, which were used as standard for the MALDI-TOF MS external calibration. Cells of *E. coli* DH5α were grown on Luria-Bertani medium agar (LB; 10 g∙L^−1^ bacto-tryptone, 5 g∙L^−1^ bactoyeast extract, 10 g∙L^−1^ NaCl) at 30 °C for 20 h. About 1 μg cellular material from a single *E. coli* DH5α colony was transferred from the LB plate to the MALDI flex plate and the matrix solution was applied as described above for the fungal analysis. All sample plates were air dried at room temperature. Each sample was spotted in duplicate to test reproducibility. During the analyses, all solutions were prepared daily and stored at 5 °C.

The analyses were performed on an Axima LNR MALDI-TOF MS system (Kratos Analytical, Shimadzu, Manchester, UK) equipped with a nitrogen laser (337 nm), where the laser intensity was set just above the threshold for ion production. Twelve defined ribosomal proteins of intact *E. coli* DH5α cells (4365.4, 5096.8, 5381.4, 6241.4, 6255.4, 6316.2, 6411.6, 6856.1, 7158.8, 7274.5, 7872.1, 9742 and 12,227.3 Da) were used as external calibrants. The mass spectra based on the mass range from 2 to 20 kDa were recorded using the linear mode with a delay of 104 ns and an acceleration voltage of 20 kV. The final spectra were generated by summing 20 laser shots accumulated per profile and 50 profiles produced per sample, which led to 1000 laser shots per summed spectrum.

The resulting peak lists were exported to the SARAMIS™ software package (Spectral Archiving and Microbial Identification System, AnagnosTec, Version 2010, Potsdam, Germany, 2010) where the final microbial identification was achieved. In SARAMIS™, peak lists of individual samples were compared on database generating a ranked list of matching spectra. This software uses a point system based on peak list with mass signals weighted according to their specificity. The similarity between individual spectra is expressed as the relative or absolute number of matching mass signals after subjecting the data to a single link agglomerative clustering algorithm. Microbial identifications by the SARAMIS™ package are based on the presence or absence of each peak in the spectra. A dendrogram of proteomic profile proximity among isolates was created using SARAMIS™ package.

### 4.8. Detection of Fumonisins by MALDI-TOF MS/MS

Samples were prepared according the previous fumonisins B1 and B2 extraction method described by Ono *et al.* [42] with some adaptation. Briefly, all of the *F. verticillioides* isolates were grown on a single-point on 6 cm PDA plates at 28 °C for 7 days. Each isolate (10^6^ conidia/mL) was then inoculated onto a 6 cm Petri dish containing ground corn moistened with distilled water (1 g/mL *v*/*v*), and autoclaved two times for 30 min at 121 °C. The cultures were incubated at 25 °C for 14 days. Three plugs of each culture samples were then extracted with 1 mL methanol-water (3:1, *v*/*v*). After 10 min incubation at room temperature, the suspension was shaken at 150 rpm for 30 min and centrifuged at 4500 *g* for 10 min.

Supernatants were then transferred to amber flasks and liquid phase was evaporated at room temperature overnight. Residues of each sample were then suspended in 0.5 mL methanol and 1 µL of each sample was mixed on a paraffin film surface with 2 µL α-cyano-4-hydroxycinnamic acid-CHCA (Fluka, Buchs, Switzerland) saturated in a solution with 33% ethanol, 33% acetonitrile, 31% H_2_O and 3% TFA. Each suspension (1 µL) was spotted onto a MALDI-TOF stainless plate (Bruker Daltonics, Billerica, MA, USA). Samples were air-dried at room temperature prior to spectral acquisition. In order to test their reproducibility samples were analysed in triplicate. In case of discordant results, analysis was repeated for at least 2 additional replicates.

During the sample preparation, all solutions were prepared and stored at 5 °C. Samples were finally analysed in a MALDI-TOF MS/MS Autoflex III (Bruker Daltonics, Billerica, MA, USA), equipped with a Nd:YAG laser at 355 nm. The spectral acquisition was assessed in the reflector positive mode, with an acceleration voltage of 19 kV under a mass range between *m*/*z* 500–4480. The external calibration was performed by using the Peptide Calibration Standard II (Bruker Daltonics).

## Figures and Tables

**Table 1:** *Fusarium verticillioides* isolates from corn kernels in different places in 3 Regions of Pernambuco State, Brazil.

Strains (Code N0: From 1 to 60)	Place	Region
1 to 17	Goiana (Itapirema)	Zona da Mata
18	Petrolina (Pedrinhas-Catingueiro)	São Francisco
19 to 30	Araripina XB 8030	Sertão
31 to 39	Araripina BRF 5036-1	Sertão
40 to 45	Araripina BRF 50361 CMS 36	Sertão
46 to 52	Araripina BRS 5036	Sertão
53 to 60	Belém do São Francisco	São Francisco
